# Pooling Bio-Specimens in the Presence of Measurement Error and Non-Linearity in Dose-Response: Simulation Study in the Context of a Birth Cohort Investigating Risk Factors for Autism Spectrum Disorders

**DOI:** 10.3390/ijerph121114780

**Published:** 2015-11-19

**Authors:** Karyn Heavner, Craig Newschaffer, Irva Hertz-Picciotto, Deborah Bennett, Igor Burstyn

**Affiliations:** 1 Department of Environmental and Occupational Health, Dornsife School of Public Health, Drexel University, Philadelphia, PA 19104, USA; E-Mail: igor.burstyn@drexel.edu; 2 A.J. Drexel Autism Institute, Dornsife School of Public Health, Drexel University, Philadelphia, PA 19104, USA; E-Mail: cjn32@drexel.edu; 3 Department of Public Health Sciences, University of California at Davis, Davis, CA 95616, USA; E-Mails: ihp@phs.ucdavis.edu (I.H.-P.); dhbennett@phs.ucdavis.edu (D.B.); 4 Department of Epidemiology and Biostatistics, Dornsife School of Public Health, Drexel University, Philadelphia, PA 19104, USA

**Keywords:** pooling, autism spectrum disorders, measurement error

## Abstract

We sought to determine the potential effects of pooling on power, false positive rate (FPR), and bias of the estimated associations between hypothetical environmental exposures and dichotomous autism spectrum disorders (ASD) status. Simulated birth cohorts in which ASD outcome was assumed to have been ascertained with uncertainty were created. We investigated the impact on the power of the analysis (using logistic regression) to detect true associations with exposure (X_1_) and the FPR for a non-causal correlate of exposure (X_2_, r = 0.7) for a dichotomized ASD measure when the pool size, sample size, degree of measurement error variance in exposure, strength of the true association, and shape of the exposure-response curve varied. We found that there was minimal change (bias) in the measures of association for the main effect (X_1_). There is some loss of power but there is less chance of detecting a false positive result for pooled compared to individual level models. The number of pools had more effect on the power and FPR than the overall sample size. This study supports the use of pooling to reduce laboratory costs while maintaining statistical efficiency in scenarios similar to the simulated prospective risk-enriched ASD cohort.

## 1. Introduction

In autism etiologic research, as in other areas of perinatal research and epidemiologic investigation, there is growing interest in analytic methods that maximize accuracy while minimizing costs. One of the methods under consideration to conserve scarce resources is pooling of bio-samples prior to laboratory analysis to conserve biological specimens, enable the study of more biomarkers (exposures), minimize problems related to the limits of detection, reduce the impact of measurement error in exposure variables, and include participants who contributed only a small quantity of bio-samples [1,2,3,4,5,6]. Pooling may also be used to screen batches of samples for an infectious agent or biomarker after which all specimens in a positive pool are tested individually [7]. Pooling (combining bio-samples from multiple individuals and analyzing them as a single sample) was proposed for public health research and surveillance in 1943 [8] and has been receiving increased attention in the epidemiology literature in the context of non-communicable diseases since 2012 [3,9,10,11,12,13,14,15,16,17,18]. The use of pooling has been investigated in other areas of study, such as perinatal epidemiology [3,19], infectious disease epidemiology [8,20,21,22,23,24], environmental epidemiology [3,10,25], cancer epidemiology [12], and genetics [10,12] but has only recently been studied in the context of research on autism spectrum disorders (ASD) [17,18]. An example of a current research area that may greatly benefit from pooling is the potential role of polychlorinated biphenyls (PCBs) in the etiology of ASD because the levels of PCBs are low, laboratory analyses are very costly, and the blood volumes needed to achieve acceptable sensitivity of analysis are large [26,27].

In applications of the pooling approach, earlier work suggests that a smaller pool size (number of participants per pool ~5) is ideal as it yields the most accurate study results but scarce resources may necessitate larger pools (e.g., with 10 or more individuals per pool), which have received less attention in the literature. Furthermore, a wide range of pooling strategies in the context of epidemiological analyses were presented by Saha-Chaudhuri *et al.*, highlighting that optimal allocation of the number of pools and number of subjects per pool should be informed by the specific confounders and effect modifiers under consideration [3,28]. Little is known about the impact of pooling in the presence of misspecification of the disease risk function (e.g., when investigators assume that there is a monotonic gradient in log(risk) when in fact there is non-linearity), miss-measured covariates, and in the presence of outcome misclassification. Here we present results of a simulation analysis designed to shed light on the effects of pooling and measurement error that is informed by interim data from the Early Autism Risk Longitudinal Investigation (EARLI). EARLI is an enriched-risk pregnancy cohort (mothers of a child affected by an ASD enrolled at the start of a subsequent pregnancy) intended to generate evidence on environmental exposures important in the etiology of ASD (http://www.earlistudy.org/) [29]. Our previous work that was informed by the EARLI elucidated the potential impact of measurement errors in exposures and outcomes under different scenarios of study size, precision of exposure measurement and magnitude of the true association [30]. We also evaluated the influence of categorization of a mismeasured exposure in this context with particular attention to non-linearity of the true association [31]. Importantly, EARLI investigators plan to conduct analyses focused on continuous phenotypic measures as well as dichotomous outcomes. Quantitative phenotypes are being increasingly considered in autism etiologic research (such as in references [32,33,34]) and may prove to be important in the study of environmental risk factors. Here we consider the same general scenarios involving both dichotomous outcomes based on categorization of a quantitative measure as in our previous paper as well as pools of different sizes and aim to determine the potential effects of pooling on power, false positives, and bias of the estimated associations between hypothetical environmental exposures and dichotomous ASD status. We also extend our previous work by considering the utility of pooling when the true shape of the exposure-response association is unknown and categorization of exposures is deemed undesirable.

## 2. Material and Methods 

### 2.1. Simulated Population

The population and sample parameters are listed in Table 1 and are based on the synthetic population used in our previous work [30,31]. Briefly, we generated a cohort of 1,000,000 children with observed sex (Z) and mismeasured gestational age (W_ga_) distributions similar to the general population in the US, accounting for shortened gestation, on average, among boys [35]. We assumed that the cohort was also exposed to two agents: exposure (1), represented by X_1_, and exposure (2), represented by X_2_, that both follow standard normal distributions and are correlated (Pearson ρ = 0.7). We posited that only exposure (1) (with values of X_1_) exerts causal influence on ASD-related phenotype. We simulated true gestational age X_ga_ (in weeks) in the cohort to follow a distribution that is (43 − χ^2^(3)), after which boys were assigned gestational age that was one week shorter for 5% of boys. The simulated population was created and all analyses were conducted using SAS version 9.2 (SAS Institute, Cary, NC, USA).

### 2.2. Covariate Measurement Error

We assumed that the ith environmental exposure (here: i = 1 or 2) is observed with classical measurement error: W_i_ = X_i_ + ε_i,_ where ε_i_~N(0,σ^2^_i_). Error was added to the individual level values as opposed to after pool allocation, implying that errors arise not from the pooling procedure in the laboratory or laboratory testing errors but from factors such as variation in the etiologically relevant time window when exposure was ascertained and random day-to-day variability in dose. The extent of measurement error for exposure 1 (ME_1_) was selected to span the plausible range: from good precision of environmental measurements with error 6% of true exposure variability to poor precision with error variance equal to the true exposure variability. Gestational age was subject to two sources of error: (a) classical error in the observed continuous measure of the length of gestation, W_ga_ = X_ga_ + ε_ga,_ where ε_ga_~N(0,σ^2^_ga_) and (b) round-off error due to reporting gestational age in completed weeks between 23 and 43 weeks. All errors were conditionally independent of each other. Specific values used in the simulation studies are given in Table 1. 

**Table 1:** True and observed values in the simulated population.

Values in the Population (Notation)	True Values	Observed Values	Measurement Error	Postulated True Association with Latent Measure of Outcome ^a^	Cutoff Used for Dichotomization
Environmental exposure 1 (X_1_)	X_1_~N(0,1), correlated with X_2_ by Pearson correlation ρ = 0.7	W_1_ = X_1_ + ε_1_	ε_1_~N(0,σ^2^), where σ^2^ ∈ {0.0625, 0.25, 1}	{0.15, 0.25, 0.5}	
Environmental exposure 2 (X_2_)	X_2_~N(0,1), correlated with X_1_ by Pearson correlation ρ = 0.7	W_2_ = X_2_ + ε_2_	ε_2_~N(0,0.25)	0	
Sex (Z)	Z~Binomial(0.5, 1)	Z	None	1	
Gestational age (X_ga_)	X_ga_ ~ (43 – χ^2^(3)) 1 week was subtracted from the above gestational age for 5% of males	W_ga_ = R((Wga + ε_ga_); 23, 43), Where R(.) is a function that is the rounded expression to integers, and then truncated to 23 to 43 weeks.	$\varepsilon_{ga}\sim N(0,\frac{1}{72})$	0.1	
Autism endophenotype (latent, Y)	ε_y_~N(0,1) Linear model Y_L_ = β_1_X_1_ + β_3_Z + β_4_Xga + ε_y_, Semi-linear model 1: threshold model If x_1_ < −1 then Y_T_ = β_3_Z+ β_4_Xga + ε_y_ If x_1_ ≥ −1 then Y_T_ = 1.5 × β_1_X_1_ + β_3_Z + β_4_Xga + ε_y_ Semi-linear model 2: saturation model If x_1_ <−1 then Y_S_ =1.5 × β_1_X_1_ + β_3_Z + β_4_Xga + ε_y_ If x_1_ ≥ −1 then Y_S_ = 0.5 × β_1_X_1_ + β_3_Z + β_4_Xga + ε_y_,	Y^*^ = R(T(y); 0, 18), where T(.) is a function that is transformed to the Y log-normal distribution that match observed AOSI in EARLI	due to rounding by R(.) ^b^	Not applicable	0–6, 7–18

Notes: AOSI—Autism Observation Scale for Infants; EARLI—Early Autism Risk Longitudinal Investigation; ^a^ coefficients of linear regression, see text and bottom of the table for details, β’s; ^b^ R(f(.); min, max) is the function that rounds values of function f(.) to integers and truncates values (retains only values) that fall within interval [min, max].

### 2.3. Linear and Semi-Linear Risk Models

We assumed that in addition to X_1_, sex (sex ratio of 4 males: 1 female [36]) and gestational age [24,37,38] exert causal influence on dichotomous ASD status and continuous ASD-related phenotype (Y). We verified that these yielded parameter estimates within the expected range. As the true shape of the exposure-response curve is unknown, the simulated disease was modeled using the following three forms:

**Linear Model**:

Y_L_ = β_1_X_1_ + β_2_X_2_ + β_3_Z + β_4_X_ga_ + ε_y_

**“Threshold” (Semi-Linear) Model**:

If x_1_ < mean_x1_-standard deviation_x1_ then Y_T_ = 0 × β_1_X_1_ + β_2_X_2_ + β_3_Z + β_4_X_ga_ + ε_y_

If x_1_ ≥ mean_x1_-standard deviation_x1_ then Y_T_ = 1.5 × β_1_X_1_ + β_2_X_2_ + β_3_Z + β_4_X_ga_ + ε_y_

**“Saturation” (Semi-Linear) Model**:

If x_1_ < mean_x1_-standard deviation_x1_ then Y_S_ = 1.5 × β_1_X_1_ + β_2_X_2_ + β_3_Z + β_4_X_ga_ + ε_y_

If x_1_ ≥ mean_x1_-standard deviation_x1_ then Y_S_=0.5 × β_1_X_1_ + β_2_X_2_ + β_3_Z + β_4_X_ga_ + ε_y_,

The deviation from linearity is created here by altering the slope of the causal association with X_1_ above and below the inflection point defined by (mean_x1_-standard deviation_x1_). Constant multipliers (here: 0.5, 1, and 1.5) are used. Thus, in the saturation model, the effect of X_1_ is 3 times greater below than above the inflection point because 1.5 × β_1_X_1_/ 0.5 × β_1_X_1_ = 3. Likewise, in the threshold model, the effect of X_1_ is 1.5 times greater above than below the inflection point because 1.5 × β_1_X_1_/β_1_X_1_ = 1.5. These multipliers were chosen arbitrarily and yet have numerical values that yield plausible “average” associations if the linear model was fitted to the data. For all models ε_y_~N(0,1) and β_2_ was 0 since X_2_ had no true effect on Y. The latent values of ASD-related phenotype (Y_L, T, S_) were computed for each of three β_1 ∈_ {0.15, 0.25, 0.5} that correspond to weak, moderate and strong associations as judged by expected odds ratios (eOR_1_) that result if X_1_ and Y_L_ are dichotomized at one standard deviation above their respective means, *i.e.* eOR_1_ = 1.5, 2, and 4.

### 2.4. Case Definition and Outcome Misclassification

The outcome measure simulated for this particular work was the Autism Observation Scale for Infants (AOSI) score that takes on values of 0–18 [39]. Latent continuous ASD-related endophenotype, was subjected to rounding error and categorized using a cutoff suggested by prior work (Zwaigenbaum L., personal communications). For Y_L_, Y_T_ and Y_S_, $Y_{C}^{*}=0$ if Y^*^ is 0–6 and $Y_{C}^{*}=1$ if Y^*^ is 7–18 (Table 1). For all exposure-disease models, subjects with a high AOSI score (at least 7) were considered to be cases. It must be noted that we do not here equate high AOSI with clinical diagnosis of ASD but merely state that ASD is a collection of traits that naturally occur on a continuous scale and are segregated into binary disease and healthy groups based on some criteria. This is consistent with the current conceptualization of ASD as a “spectrum” of phenotypes, not a definitive state common to all cases, as would be true of another condition such as death from a cardiac event or acquisition of an infection. However, diagnostic thresholds are considered essential in clinical practice as well as epidemiology and the chosen cutoff of 7 has been suggested to have clinical and/or etiologic significance. One can argue that various AOSI sub-scales may be more related to environmental exposure than others but this is not our focus here and our argument applies to any measures of continuous traits that have AOSI-like properties.

### 2.5. Pool Construction and Composition and Simulated Cohorts

**Figure 1:**
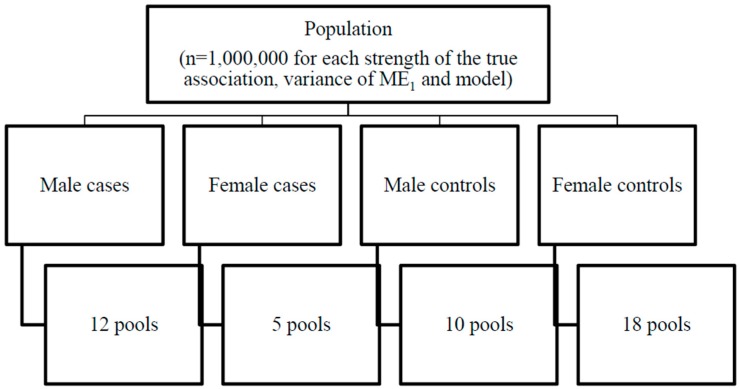
Strata and pool allocation. ME_1_—measurement error variance for the causal exposure; Sample size 225—5 individuals per pools; Sample size 450—10 individuals per pools; Sample size 675—15 individuals per pools.

Strata (based on sex and case status) and pool allocation are illustrated in Figure 1. For each model, strength of the true association of X_1_, variance of ME_1_, and shape of the exposure-disease relationship, members of the population were divided into 4 strata based on the value of their dichotomous AOSI score (high *versus* low) and sex, similar to the pooling strategy suggested by Weinberg and Umbach [6]. Each stratum was divided into pools. One thousand cohorts (*i.e*., study samples) each of size (n) 225, 450 and 675 were randomly selected with 5, 10 and 15 individuals per pool (g will be used to refer to the pool size), respectively. The pools were allocated as follows: 10 pools for male controls, 18 pools for female controls, 12 pools for male cases and five pools for female cases. Many pool allocation schemes are possible but this scheme reflects a plausible situation that would arise when stratifying on an established strong risk factor (e.g., sex) and maintaining pools of uniform size within a study. In this scheme, the overall cost of laboratory analyses, which is determined by the number of pools, is kept constant across simulations, *i.e.*, reflecting the reality of having to conduct a study on a fixed budget.

### 2.6. Assessing the Effect of Pooling

The pooled models were in the form of:

$$LOGIT(pr({Y_{cpool}}^{*}=1))=c_{o}+c_{1}\sum W_{1}+c_{2}\sum W_{2}+c_{3}\sum Z+c_{4}\sum W_{ga}$$

with offset = ln(rg_z_) where $rg_{z}=\frac{\#\;case\;pools\;of\;size\;g\;and\;sex\;z}{\#\;control\;pools\;of\;size\;gand\;sex\;z}$.

Thus, for logistic models, exp(c_1_) is the odds ratio of the estimated effect of X_1_ (OR_1_) on odds of AOSI ≥ 7 and exp(c_2_) is the odds ratio of the observed effect of X_2_ (OR_2_) on odds of AOSI ≥ 7. All independent variables except for z were considered as continuous predictors of the dichotomized AOSI score. We also fitted analogous logistic regression models with values of exposure that were not contaminated by measurement error, e.g., with X_1_ instead of W_1_, *etc.* These models were repeated tor Y_T_ and Y_S_. The pooled models were compared to the individual level population and replicate models in the form:

$$LOGIT(pr(Y_{c}^{*}=1)=c_{0}+c_{1}X_{1}+c_{2}X_{2}+c_{3}Z+c_{4}X_{ga}(logistic)$$

Regression models that did not converge or with an OR_1_ or OR_2_ less than or equal to 0.1 or at least 10 were considered unstable and not included in bias calculations.

### 2.7. Comparison of the Replicates to the Population

We compared the replicate results to the population results to get an idea of how close pooled estimates are to the true parameter values. For each combination of model, strength of the true association, variance of ME_1_, and sample (or pool) size, the mean OR, and the power of the analysis and bias in the OR for exposure 1 were calculated for the models without measurement error. These quantities as well as the false positive rate (FPR) and bias in the OR for exposure 2 were calculated for the models with measurement error. 

The numerator for the power and FPR calculation was the number of models for which OR_1_ and OR_2_, respectively, were 0.1 or less, at least 10, or statistically significant (α=0.05). The bias was calculated as:

$$bias_{OR_{W}}=\frac{\sum_{k=1}^{\#\;replicates}\frac{{\overset{⌢}{OR}}_{W_{k}}}{OR_{Xpopulation}}}{\#\;stable\;replicates}$$

for replicate ORs between 0.1 and 10. The percent mean difference in OR between the pooled and individual level replicate analyses was calculated for X_1_ and X_2_.

### 2.8. Comparison of the Individual Level Replicate Analysis and Pools of Different Sizes

A comparison of the pooled results to the individual level replicate results gives us an idea of what information is lost due to pooling. For each replicate of sample size 675 with a linear exposure-disease relationship, individual level (g = 1) and pooled analyses were compared. Pools of size 5 and 15 were analyzed for all replicates. This represents scenarios where the cost of analysis increases for a fixed cohort size as the pools become smaller. The power, FPR, and bias in OR_1_ and OR_2_ were compared.

## 3. Results

**Figure 2:**
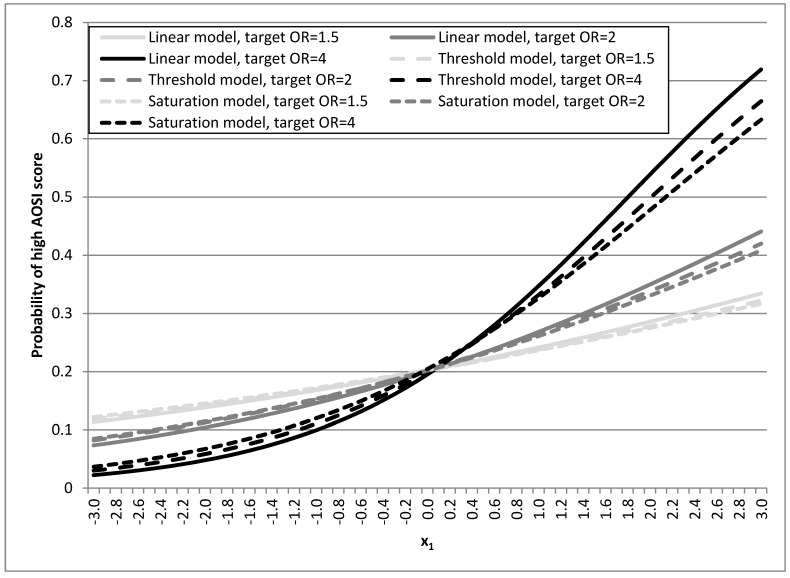
Distribution of high AOSI scores in the linear, threshold and situation models. AOSI—Autism Observation Scale for Infants.

The characteristics of the viable population are similar to what was expected based on the simulation parameters (data not shown). The *logit* plots of the linear, threshold and saturation models appear to be very similar despite the fact that they have potentially very different biological implications (Figure 2). Unless otherwise stated, the description of the results pertains to the linear model.

The proportion of the replicates for which there was a stable logistic regression model, which converged within 50 iterations and yielded an OR_1_ and OR_2_ between 0.1 and 10 (not inclusive), drastically decreased between the moderate effect (eOR = 2: when nearly all the replicates produced a stable regression model) and strong effect (eOR = 4) and decreased with increasing sample size (Table S1). There were a greater number of stable regression models for the semi-linear models than the linear models when the eOR was 4. Overall, the models with greater measurement error had a higher proportion of stable regression models. The characteristics of the unstable models for pool size 15, eOR = 4 and the smallest measurement error variance are in Table S2. (This scenario was chosen as it had the greatest number of unstable replicates.) Nearly all of these unstable replicates had OR_1_ ≥ 10 and half of these also had OR_2_ ≥ 10. 

**Figure 3:**
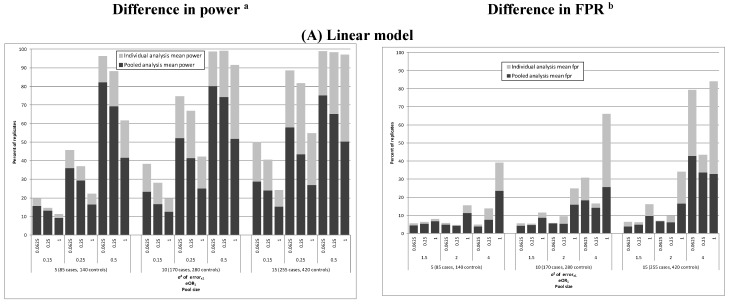

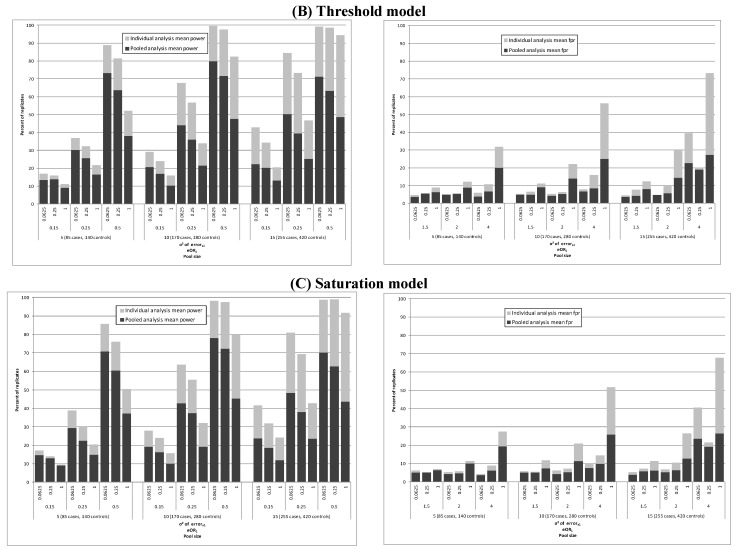
Mean differences in power and FPR due to pooling in 1000 replicates for models with measurement error. eOR—expected odds ratio, FPR—false positive rate; OR—odds ratio: (^a^) $Power=\frac{\#\;\mathrm{mod}els\;with\;OR_{x_{1}}\;\leq \;0.1\;or\;OR_{x_{1}}\geq \;10\;or\;significant\;OR_{x_{1}}}{Total\;\#\;of\;replicates}$; (^b^) $FPR=\frac{\#\;\mathrm{mod}els\;with\;OR_{x_{2}}\leq \;0.1\;or\;OR_{x_{2}}\geq \;10\;or\;significant\;OR_{x_{2}}}{Total\;\#\;of\;replicates}$.

We next compared results of the pooled replicate analysis with the individual level population analysis and individual level replicate analysis. There was a loss of power for many of the pooled models: e.g., the power was 25%–50% for the cohort of size 450 and eOR of 2, but little difference in the OR_1_ (all bias terms between 0.8 and 1.2 except in cases of strong true association or large variance in measurement error) (Table 2, Figure 3). For all combinations of pool size and strengths of the true association, except eOR = 4, there was an increase in power as the pool size (and sample size) increased. The FPR was relatively low for the pooled analysis: less than 15% unless there was a strong association (eOR = 4), or a moderate association (eOR = 2) and large variance in measurement error. Likewise, the mean OR_2_ (*i.e*., bias in the estimate for the non-causal exposure) was low (1.0–1.1 in most of the scenarios) except in the models with large measurement error and strong true association (eOR = 4), when mean estimates of OR_2_ were 1.4–1.6 (data not shown). In general, the power for detecting associations of X_1_ and W_1_ was slightly lower for the semi-linear models than the linear models but the bias in the estimate of the overall “average effect” was similar (Figure 3). However, for the strong true association, large measurement error variance and, especially, bigger pool size, the semi-linear models had higher power than the linear models.

**Table 2:** Performance of pooled logistic regression analysis: averages of 1000 simulation realizations for each scenario with a consistent number of pools (45).

Cohort (Pool) Size	eOR_1_ (eβ_1_)	${\mathrm{ME}}_{X_{1}}$ (σ^2^)	Comparison to Population Analysis			Comparison to Individual Level Replicate Analysis	
			Exposure 1		Exposure 2	% Mean Change in OR ^d^	
			Power ^a^ (%)	Bias ^b^	FPR ^c^ (%)	Exposure 1	Exposure 2
**Linear model**							
225 (5)	1.5 (0.15)	Truth ^e^	26.3–27.5	1.1		3.1–3.6	
		0.0625	15.7	1.1	4.4	3.2	0.0
		0.25	13.1	1.0	5.4	2.7	0.2
		1	9.3	0.9	6.8	1.2	1.1
	2.0 (0.25)	Truth ^e^	60.8–63.3	1.1		5.7–6.5	
		0.0625	36.0	1.1	4.8	6.1	−0.8
		0.25	29.4	1.0	4.2	4.3	0.5
		1	16.6	0.8	11.4	2.4	2.4
	4.0 (0.5)	Truth ^e^	98.7–98.9	1.3		10.6–12.3	
		0.0625	82.1	1.3	3.9	13.6	0.2
		0.25	69.3	1.0	7.6	10.8	1.9
		1	41.5	0.7	23.6	6.0	4.0
450 (10)	1.5 (0.15)	Truth ^e^	40.3–41.6	1.1		3.7–4.1	
		0.0625	23.3	1.1	4.2	3.9	−0.7
		0.25	16.8	1.0	4.7	2.9	0.5
		1	12.5	0.9	8.7	1.9	1.5
	2.0 (0.25)	Truth ^e^	82.0–83.4	1.1–1.2		7.8–8.6	
		0.0625	52.1	1.1	5.5	8.3	−1.0
		0.25	41.4	1.0	5.3	7.2	0.3
		1	25.0	0.8	15.8	3.6	3.9
	4.0 (0.5)	Truth ^e^	92.9–93.6	1.4		16.6–18.3	
		0.0625	80.2	1.3	18.4	16.2	−0.7
		0.25	74.2	1.1	14.2	16.6	2.0
		1	51.8	0.7	25.7	10.1	6.6
675 (15)	1.5 (0.15)	Truth ^e^	48.3–50.5	1.1		4.9–5.0	
		0.0625	28.8	1.1	3.9	5.4	−0.8
		0.25	24.0	1.0	4.8	5.1	−1.1
		1	15.3	0.9	9.7	2.8	1.3
	2.0 (0.25)	Truth ^e^	87.8–89.4	1.2		9.4–11.3	
		0.0625	57.9	1.2	6.7	11.2	−0.2
		0.25	43.5	1.1	6.1	9.0	0.7
		1	26.9	0.8	16.5	4.5	3.7
	4.0 (0.5)	Truth ^e^	82.2–84.9	1.3–1.4		15.3–16.8	
		0.0625	75.2	1.4	42.8	18.3	−2.9
		0.25	65.1	1.1	33.8	17.0	2.3
		1	28.8	0.7	12.2	0.1	0.1
**Threshold model**							
225 (5)	1.5 (0.15)	Truth ^e^	22.6–24.4	1.1		2.9-3.3	
		0.0625	13.4	1.1	3.6	2.9	−0.1
		0.25	13.9	1.0	5.6	2.8	−0.1
		1	9.1	0.9	6.3	1.2	0.4
	2.0 (0.25)	Truth ^e^	50.8–54.4	1.1		4.3–4.8	
		0.0625	30.1	1.1	5.0	5.0	−0.9
		0.25	25.7	1.0	5.3	4.2	0.1
		1	16.5	0.9	8.9	2.4	1.8
	4.0 (0.5)	Truth ^e^	96.4–96.9	1.2–1.3		7.8-8.8	
		0.0625	73.2	1.2	3.8	9.4	−0.9
		0.25	63.8	1.0	6.8	7.9	0.8
		1	38.1	0.7	20.1	4.4	3.3
450 (10)	1.5 (0.15)	Truth ^e^	36.5–36.7	1.1		3.4–3.6	
		0.0625	20.6	1.1	4.8	3.1	0.0
		0.25	17.0	1.0	4.9	2.9	0.4
		1	10.3	0.9	9.0	1.7	1.2
	2.0 (0.25)	Truth ^e^	73.5–76.2	1.1		6.1–6.9	
		0.0625	44.0	1.1	4.3	6.6	0.3
		0.25	35.9	1.0	5.3	6.0	0.9
		1	21.5	0.8	14.1	3.2	2.2
	4.0 (0.5)	Truth ^e^	95.0–97.1	1.2–1.3		10.8–11.5	
		0.0625	79.8	1.2	6.7	12.7	−0.8
		0.25	71.6	1.0	8.4	11.2	1.6
		1	47.5	0.7	25.1	6.8	5.2
675 (15)	1.5 (0.15)	Truth ^e^	42.9–43.9	1.1		4.2–4.6	
		0.0625	22.3	1.1	3.7	4.9	−1.0
		0.25	20.2	1.0	4.3	3.9	−0.5
		1	13.2	0.9	8.0	2.0	1.2
	2.0 (0.25)	Truth ^e^	80.8–83.1	1.2		7.5–8.5	
		0.0625	50.3	1.1	4.6	8.4	−0.6
		0.25	39.5	1.0	5.8	6.6	1.4
		1	25.3	0.9	14.5	3.8	3.5
	4.0 (0.5)	Truth ^e^	85.0–86.9	1.3		12.2–13.3	
		0.0625	71.2	1.3	22.8	15.8	−3.5
		0.25	63.4	1.0	19.0	11.8	3.8
		1	48.7	0.8	27.3	8.3	5.9
**Saturation model**							
225 (5)	1.5 (0.15)	Truth^e^	19.4–22.5	1.0–1.1		2.3–3.5	
		0.0625	14.6	1.1	5.2	3.4	0.1
		0.25	12.9	1.0	5.1	2.8	−0.8
		1	9.0	0.9	6.3	0.8	−0.1
	2.0 (0.25)	Truth ^e^	49.7–52.7	1.1		4.7–5.4	
		0.0625	29.4	1.1	4.3	4.7	−0.2
		0.25	22.6	1.0	4.6	3.8	0.1
		1	14.9	0.9	10.1	2.5	1.9
	4.0 (0.5)	Truth ^e^	95.5–96.1	1.2		10.1–10.6	
		0.0625	70.7	1.2	3.6	10.6	−0.1
		0.25	60.4	1.0	6.2	8.8	0.5
		1	37.3	0.7	19.5	4.6	3.4
450 (10)	1.5 (0.15)	Truth ^e^	32.9–33.3	1.1		3.4–4.1	
		0.0625	19.1	1.1	5.2	3.6	−0.6
		0.25	16.3	1.0	5.1	3.4	−0.1
		1	10.0	0.9	7.3	1.7	1.4
	2.0 (0.25)	Truth ^e^	72.2–73.8	1.1		6.7–7.0	
		0.0625	42.8	1.1	4.3	6.5	0.7
		0.25	37.4	1.0	5.4	5.4	1.2
		1	19.2	0.9	11.3	3.1	2.9
	4.0 (0.5)	Truth ^e^	95.3–96.6	1.4		15.9–17.7	
		0.0625	78.1	1.3	7.6	16.8	−0.4
		0.25	72.2	1.1	9.8	14.6	2.7
		1	45.4	0.8	25.8	7.6	5.8
675 (15)	1.5 (0.15)	Truth ^e^	41.3–41.8	1.1		4.6–5.3	
		0.0625	23.7	1.1	3.8	5.6	−1.1
		0.25	18.6	1.0	5.7	4.1	0.3
		1	11.9	0.9	6.2	2.4	1.3
	2.0 (0.25)	Truth ^e^	78.7–82.6	1.1–1.2		8.9–9.3	
		0.0625	48.4	1.2	5.4	10.3	−1.7
		0.25	38.0	1.0	6.4	7.1	1.1
		1	23.6	0.9	12.8	4.2	3.4
	4.0 (0.5)	Truth ^e^	85.4–87.3	1.4		15.5–18.2	
		0.0625	70.2	1.4	23.6	17.5	−1.7
		0.25	62.6	1.1	19.2	14.3	4.4
		1	43.7	0.8	26.5	10.5	7.9

Notes: eOR—expected odds ratio; FPR—false positive rate; ME—measurement error; OR—odds ratio; (^a^) $Power=\frac{\#\;\mathrm{mod}els\;with\;OR_{x_{1}}\;\leq \;0.1\;or\;OR_{x_{1}}\;\geq \;10\;or\;significant\;OR_{x_{1}}}{Total\;\#\;of\;replicates}$; (^b^) $Bias=\frac{\sum_{k=1}^{1000}(\frac{OR_{xk\;pooled}}{OR_{x\;population}})}{\#\;\;of\;stable\;replicates}where\;0.1<OR_{x_{1}k}<10$ (^c^) $FPR=\frac{\#\;\mathrm{mod}els\;with\;OR_{x_{2}}\leq \;0.1\;or\;OR_{x_{2}}\geq \;10\;or\;significant\;OR_{x_{2}}}{Total\;\#\;of\;replicates}$ (^d^) $\%\;mean\;change\;in\;OR=100*\frac{\sum_{k=1}^{1000}(\frac{OR_{xk\;pooled}-OR_{xk\;individual}}{OR_{xk\;pooled}})}{\mathrm{Total}\;\#\;\mathrm{of}\;\mathrm{replicates}}where\;0.1<OR_{xk\;pooled}<10$; (^e^) Models without measurement error. Values vary as a different viable population was selected for each measurement error scenario.

### Comparison of Pool (g) and Sample Sizes (n)

When the pool and cohort size varied, a number of trends emerged (Table 2). Larger cohort and pool sizes (n ≥ 450 with g ≥ 10) had more power to detect a relationship than smaller pool sizes for smaller true effects with the linear model (e.g., for moderate ME_1_ and eOR = 2, power = 41%–44% for n = 450 or 675 and 29% for n = 225). 

**Table 3:** Comparison of linear model, individual level and pool sizes of 5 and 15 for the same replicates (linear model only, cohort size = 675).

eOR_1_ (eβ_1_)	ME_1_ (σ^2^)	Individual Level Analysis (Pool Size 1)				Pool Size 5				Pool Size 15			
		Stable Replicates ^a^	Exposure 1		Exposure 2	Stable Replicates ^a^	Exposure 1		Exposure 2	Stable Replicates ^a^	Exposure 1		Exposure 2
			Power ^b^	Bias ^c^	FPR ^d^		Power ^b^	Bias ^c^	FPR ^d^		Power ^b^	Bias ^c^	FPR ^d^
1.5 (0.15)	Truth ^e^	1000	97.5–98.4	1.0		997–999	92.0–93.8	1.1		997–999	48.3–50.5	1.1	
	0.0625	1000	90.5	1.0	52.8	992	84.1	1.1	54.8	992	28.8	1.1	3.9
	0.25	1000	86.3	0.9	53.8	994	79.0	1.0	57.1	994	24.0	1.0	4.8
	1	1000	75.6	0.9	66.6	994	70.5	0.9	61.7	994	15.3	0.9	9.7
2.0 (0.25)	Truth ^e^	1000	100.0	1.0		967–977	99.7–99.8	1.2		967–977	87.8–89.4	1.2	
	0.0625	1000	99.2	1.0	55.4	947	96.5	1.2	57.5	947	57.9	1.2	6.7
	0.25	1000	99.0	0.9	62.4	970	94.0	1.1	63.1	970	43.5	1.1	6.1
	1	1000	93.2	0.8	82.0	978	84.9	0.8	75.1	978	26.9	0.8	16.5
4.0 (0.5)	Truth ^e^	1000	100.0	1.0		522–537	100.0	1.3–1.4		522–537	82.2–84.9	1.3–1.4	
	0.0625	1000	100.0	0.9	53.1	458	99.7	1.4	68.9	458	75.2	1.4	42.8
	0.25	1000	100.0	0.8	75.4	603	98.2	1.1	71.6	603	65.1	1.1	33.8
	1	1000	100.0	0.6	99.5	769	96.0	0.7	88.9	769	28.8	0.7	12.2

Notes: eOR—expected odds ratio; FPR—false positive rate; ME_1_—measurement error variance for exposure 1; OR—odds ratio; (^a^) Number of replicates for which the OR_x1_/OR_w1_ and OR_w2_ were between 0.1 and 10. (^b^) $Power=\frac{\#\;\mathrm{mod}els\;with\;OR_{x_{1}}\;\leq \;0.1\;or\;OR_{x_{1}}\geq \;10\;or\;significant\;OR_{x_{1}}}{Total\;\#\;of\;replicates}$; (^c^) $Bias=\frac{\sum_{k=1}^{1000}(\frac{OR_{xk\;pooled}}{OR_{x\;population}})}{\#\;\;of\;stable\;replicates}where\;0.1<OR_{x_{1}k}<10$; (^d^) $FPR=\frac{\#\;\mathrm{mod}els\;with\;OR_{x_{2}}\leq \;0.1\;or\;OR_{x_{2}}\geq \;10\;or\;significant\;OR_{x_{2}}}{Total\;\#\;of\;replicates}$; (^e^) Models without measurement error. Values vary as a different viable population was selected for each measurement error scenario.

This was also true for the threshold and saturation models. However, for the stronger true effect (eOR = 4) and moderate–to-high measurement error variance the power was greatest for pool size 10 (n = 450) for the linear and semi-linear models. When there was no measurement error, power increased with increasing pool sample size for small and moderate true associations and decreased with increasing sample size when the true effect of X_1_ was large. In general, larger sample size resulted in a slight increase in FPR for a moderate true effect (FPR = 4%, 5% and 6% for eOR = 2 and moderate ME_1_). For small and large eOR the relationship between FPR and pool and sample size was more complex.

A comparison of the linear model with cohort size of 675 analyzed at the individual level and using pools of size 5 and 15 is shown in Table 3. For more modest eORs (1.5 or 2), the results of the individual logistic regression and analysis of pools of size 5 were very similar. There was a dramatic drop in power and the FPR between pool sizes of 5 and 15. The bias was identical for the two pool sizes. However, for a stronger true association (eOR = 4) and smaller measurement error variance, the bias in OR_1_ was in the opposite direction compared to the individual level models. The bias in the association of the miss-measured non-causal exposure (mean OR_2_) increased slightly when the pool size changed from 1 (individual level analysis) to 5 but there was no difference between pool sizes of 5 and 15 (data not shown). The number of replicates for which a logistic model was stable was the same for pool sizes 5 and 15. 

## 4. Discussion

This study supports the use of pooling as an efficient means to reduce laboratory costs in scenarios similar to this specific prospective risk-enriched ASD cohort that aims to elucidate environmental associations (EARLI). The results of this series of simulations found that there is some loss of power for pooled compared to individual level models. We also quantified the resulting reduction in the false positive rate between pool sizes of 5 and 15 when the cohort size was held constant. In addition, there was minimal change in the measure of association for the main effect (X_1_). This was true for the linear as well as semi-linear models. Consistent with previous work [30], the models with measurement error had lower power than models without measurement error.

We looked at larger pool sizes than many of the previous articles about pooling methods and simulations (e.g., [3,12,28]). Saha-Chaudhuri [28] and Weinberg [3] found that pooling had little effect on power with large sample sizes (but did not consider pool sizes larger than (6) and recommended smaller pool sizes. Small pool sizes are indeed ideal in many circumstances but as Dorfman observed in 1943, financial constraints may have an effect on the feasible pool sizes and number of pools [8]. Pool size had little effect on bias but greatly affected the power and FPR in our simulations. In addition, we found that pool size 5 is best if it is suspected that the true association is weak and pool size of 10 may be better when a stronger true effect is hypothesized. A wide range of putative strengths of exposure-autism associations was included here to model the possibility of finding either a weak risk factor or a “smoking gun” (e.g., as exists for the four-fold increased risk of ASD for males).

We demonstrated that even pool sizes as large as 15 lead to fairly accurate results, albeit with considerable loss of power, even when measurement error is taken into consideration, in scenarios similar to those of EARLI. Furthermore, the larger pool size resulted in a much lower FPR, which may be desirable in circumstances where multiple exposures will be examined, such as research into the etiology of ASD where well-founded *a priori* hypotheses are lacking. The number of pools has more of an effect on power than the overall number of subjects. Although the loss in power for pool size of 15 makes it hard to justify looking at larger pool sizes for the sample sizes and number of pools under consideration here, investigators with considerably larger samples should consider running this set of simulations with a larger pool size. Fortunately, the measures of association are not very biased for large pool sizes, so if small pool sizes are cost prohibitive, researchers should focus more on the magnitude of these associations than on significance testing. 

It should also be noted that for these numbers of pools (and sample sizes) we were unable to stratify by more than two dichotomous variables (case status and sex). Knowledge of the determinants of exposure may help construct more efficient pooling schemes, as is done in group-based exposure assessment in occupational epidemiology [40]. However, it is not currently clear how pooling and exposure modeling or grouping can be used together.

There were a large number of replicates for which the logistic regression models did not yield a stable measure of association (OR between 0.1 and 10) when the pool size and true effect of the causal exposure were large. If a researcher has a sample such as this it should be obvious, based on the bivariate and stratified analyses, that a regression model is not appropriate. In fact, our inspection of the individual simulations revealed that in such cases, an investigator would have no doubt, based on descriptive analyses, that there is evidence to support the existence of an association (this presumed certainty of an association is the reason for their inclusion in the numerator of the power and FPR calculations). Pooling performs well even with a high degree of measurement error as long as the pool size is small and the strength of the true association is moderate. Measurement error was added to the individual values for exposure (X_1_) not to the analytical values that results after pooling of specimens. This was done based on the assumption that error occurs at the time of sampling measurement and that assay-based error is typically miniscule compared to the day-to-day variance in exposure. Weinberg and Umbach modeled both types of error and found that “if the measurement error is individual based, the dependence on g disappears and pooling has no effect on the bias in the β estimation” [6]. We found that, except in cases of a strong true association, the change in bias resulting from increasing variance in measurement error was only slightly affected by pooling (Table 3). 

Pooling may offer several advantages in scenarios similar to those in EARLI. In addition to the financial savings for analyzing fewer samples for an individual exposure, a limited amount of bio-samples can be analyzed for more environmental exposures than would be possible in an individual-level analysis. As pooling resulted in fewer spurious results (*i.e*., the FPR was lower), researchers may have more confidence in variables identified through pooled analysis. Power is important but false positives do not in any way help create useful interventions. When selecting a pooling strategy, researchers will need to balance this lower FPR against the relatively low power in some of the scenarios. Although a multitude of scenarios were studied here, we will provide our syntax upon request so that researchers who have data with other exposure distributions (including potentially skewed exposure data such as is likely with PCBs), exposure-response relationships and error distributions can investigate the ideal pool size for their study. 

## 5. Conclusions

In conclusion, our current work supports the use of pooling as a means to reduce laboratory costs while maintaining the statistical efficiency of studies that are similar to the simulated prospective cohort. Given its commendable track record under the realistic constraints of observational studies, pooling should be considered as an analytical strategy in any epidemiological study that faces constraints on the cost of analyses and availability of biological samples. 

## Supplementary Files

Supplementary File 1

## List of Abbreviations

ASD: autism spectrum disorders
PCB: polychlorinated biphenyls
AOSI: Autism Observation Scale for Infants
EARLI: Early Autism Risk Longitudinal Investigation
OR: odds ratio
FPR: false positive rate
