# Supplementary material for: Pooling Bio-Specimens in the Presence of Measurement Error and Non-Linearity in Dose-Response: Simulation Study in the Context of a Birth Cohort Investigating Risk Factors for Autism Spectrum Disorders
Source: Int J Environ Res Public Health. 2015 Nov 19;12(11):14780–99. doi: 10.3390/ijerph121114780 (PMC4661679; doi:10.3390/ijerph121114780)
Supplement: Supplementary File 1 [file ijerph-12-14780-s001.pdf]

Supplementary Information

Pooling Bio-Specimens in the Presence of Measurement Error and Non-Linearity in Dose-Response: Simulation Study in the Context of a Birth Cohort Investigating Risk Factors for Autism Spectrum Disorders

Table S1. Number of stable replicates. For poole analysis: 1000 simulation realizations for each scenario [a].

Cohort (Pool) Size	eOR1 (eβ1)	ME_{X_1} (σ^2)	Linear Model	Threshold Model	Saturation Model
225 (5)	1.5 (0.15)	Truth [b]	997–1000	1000	1000
		0.0625	998	999	1000
		0.25	1000	1000	999
		1	1000	1000	1000
	2.0 (0.25)	Truth [b]	998–999	998–1000	999–1000
		0.0625	997	999	998
		0.25	998	999	999
		1	999	1000	1000
	4.0 (0.5)	Truth [b]	951–964	979–987	987–992
		0.0625	949	970	980
		0.25	972	987	994
		1	999	999	994
450 (10)	1.5 (0.15)	Truth [b]	998–999	998–1000	999–1000
		0.0625	996	999	1000
		0.25	998	998	1000
		1	997	1000	999
	2.0 (0.25)	Truth [b]	990–996	993–1000	994–999
		0.0625	983	999	993
		0.25	993	995	994
		1	997	997	998
	4.0 (0.5)	Truth [b]	791–804	907–916	912–924
		0.0625	736	906	887
		0.25	839	929	928
		1	955	971	968
675 (15)	1.5 (0.15)	Truth [b]	997–999	997–1000	998–999
		0.0625	992	991	993
		0.25	994	996	993
		1	994	996	997
	2.0 (0.25)	Truth [b]	967–977	982–994	980–986
		0.0625	947	977	969
		0.25	970	987	979
		1	978	984	989

Table S1. *Cont.*

Cohort (Pool) Size	eOR₁ (eβ₁)	ME_{X_1} (σ^2)	Linear Model	Threshold Model	Saturation Model
675 (15)	4.0 (0.5)	Truth [b]	522–537	740–770	747–749
		0.0625	458	722	685
		0.25	603	798	769
		1	769	893	879

Notes: ([a]) Number of replicates for which the OR_{x1}/OR_{w1} and OR_{w2} were between 0.1 and 10. ([b]) Models without measurement error. Values vary as a different viable population was selected for each measurement error scenario.

Table S2. Description of 533. Unstable replicates of size 675 (g = 15), eOR = 4 and variance, ME_{x1} = 0.0625, n and percent of total [a].

		OR_{w1}							
		≤0.1		Between 0.1 and 10		≥10		Total	
		n	% of Total	n	% of Total	n	% of Total	n	% of Total
OR_{w2}	≤0.1	10	1.9	0		167	31.3	177	33.2
	Between 0.1 and 10	0		0		103	19.3	103	19.3
	≥10	4	0.7	4	0.7	245	46.0	253	47.5
	Total	14	2.6	4	0.7	515	96.6	533	100.0

Notes: ([a]) Excludes 9 replicates with convergences problems: 2 exceeded the iteration limit (50) and 7 had questionable convergence (The relative Hessian convergence criterion is greater than the limit of 0.0001).

© 2015 by the authors; licensee MDPI, Basel, Switzerland. This article is an open access article distributed under the terms and conditions of the Creative Commons Attribution license (http://creativecommons.org/licenses/by/4.0/).
